# Drug‐Induced Fuchs Syndrome With Isolated Oral Involvement: A Rare Case Report

**DOI:** 10.1155/crdm/9455957

**Published:** 2026-01-15

**Authors:** Salina Paudel, Manoj Kumar Bhatt, Shiv Kumar Chaudhary, Sandhya Silwal, Abinash Parajuli, Pramod Kumar Kafle

**Affiliations:** ^1^ Department of Internal Medicine, Tuberculosis Treatment Center, Pokhara, Gandaki, Nepal; ^2^ Department of Internal Medicine, Dadeldura Hospital, Dadeldura, Sudurpashchim, Nepal; ^3^ Department of Dentistry, College of Medical Sciences, Chitwan, Bagmati, Nepal, cmsnepal.edu.np; ^4^ Department of Internal Medicine, Manipal College of Medical Sciences, Pokhara, Gandaki, Nepal, manipal.edu.np; ^5^ Department of Internal Medicine, Chitwan Medical College, Bharatpur, Bagmati, Nepal, cmc.edu.np

**Keywords:** amoxicillin, delayed hypersensitivity, Fuchs syndrome, oral mucositis, Stevens–Johnson syndrome

## Abstract

Stevens–Johnson syndrome (SJS) is a rare, potentially life‐threatening mucocutaneous disorder characterized by epidermal necrosis and mucosal bullous lesions involving less than 10% of the total body surface area. The majority of cases are aggravated by delayed hypersensitivity reactions to medications. An uncommon presentation of SJS is isolated mucosal involvement without skin lesions, referred to as “Fuchs syndrome.” This variant is most frequently linked to Mycoplasma pneumoniae infection and certain drugs, and it often poses a diagnostic challenge due to its similarity with other mucosal pathologies. We report a case of a 6‐year‐old boy who developed isolated oral lesions following amoxicillin therapy. Prompt identification and supportive management led to complete recovery. This case emphasizes the importance of early recognition and intervention in atypical presentations of SJS.

## 1. Introduction

Stevens–Johnson syndrome (SJS) is a severe mucocutaneous hypersensitivity reaction that can be life‐threatening. It is a Type IV immune response involving the skin and mucous membranes, presenting as erythematous eruptions, erosions, and epidermal separations. Initial lesions may present as macules, papules, or nodules, which can evolve into reddish patches, vesicles, bullae, or hemorrhagic sites [1]. Clinically, SJS shares features with toxic epidermal necrolysis (TEN), but the extent of epidermal separation differentiates them: SJS involves < 10% of the body surface area, while TEN affects > 30% [2].

When mucosal involvement occurs without cutaneous lesions, the condition is termed Fuchs syndrome [3]. The differential diagnosis for isolated mucositis includes infectious causes such as oral candidiasis and herpes simplex virus (HSV); autoimmune conditions like pemphigus vulgaris, Behçet’s disease, and systemic lupus erythematosus; nutritional deficiencies such as vitamin B12 or iron deficiency; and drug‐induced mucosal injury. The clinical overlap between these disorders can make diagnosis particularly difficult in pediatric patients.

Here, we describe an unusual case of mucosal‐predominant SJS in a child, triggered by amoxicillin, who presented with painful oral ulcerations and bleeding in the absence of cutaneous lesions. Drug stoppage and supportive care led to significant clinical improvement.

## 2. Case Presentation

A six‐year‐old male presented to the Emergency Department at Kapilvastu Hospital with complaints of bleeding from the lips and ulcers in the mouth. He also reported fever and rashes around the lips for 1 week. There was no known history of trauma or similar episodes. However, the patient’s mother stated that he had taken oral amoxicillin 1 week earlier for bacterial pharyngitis. The first appearance of symptoms was noted 3‐4 days after the medication, with ulcerations and bleeding over the lips and oral cavity. Other personal and family histories were unremarkable. On arrival, his GCS was 15/15, pulse 94/min, blood pressure 100/70 mmHg, respiratory rate 23/min, and temperature 36.9°C.

**Figure 1:**
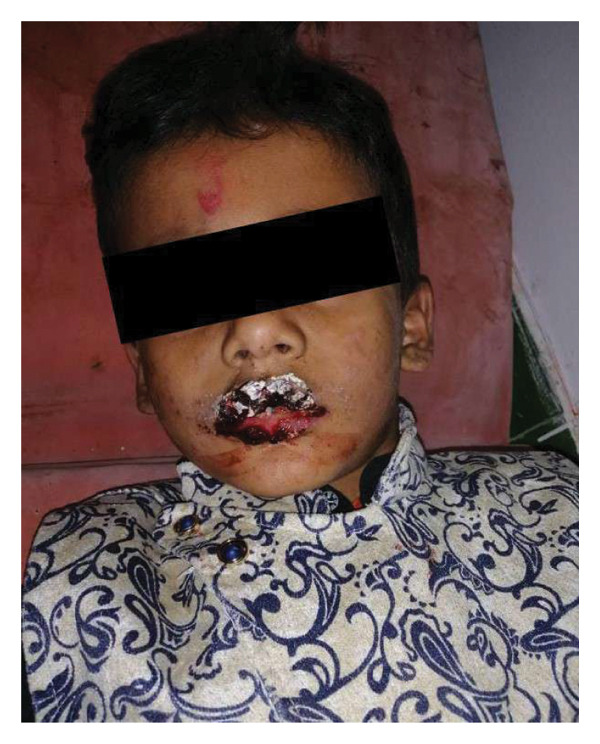
Hemorrhagic crust over lips.

On examination, there were hemorrhagic crusts over the upper and lower lips, which bled upon touch, along with erosions over the lower gums and bilateral conjunctivitis (Figure 1). No other cutaneous or mucosal lesions were identified elsewhere on the body. The findings were localized to the face and oral cavity, supporting the diagnosis of SJS with isolated oral and ocular involvement. According to the Lund–Browder chart, the total body surface area involved was approximately 5%. A clinical diagnosis of drug‐induced SJS was made.

Laboratory results revealed WBC 7300/mm^3^, RBC 5.21 million/mm^3^, platelets 7.78 lakh/mm^3^, hemoglobin 9.4 g/dL, neutrophils 37%, lymphocytes 57%, eosinophils 4%, monocytes 2%, basophils 1%, PCV 28.3%, MCV 54.4 fL, MCHC 33.2%, and MCH 18 pg. Histopathology was not available at our hospital. The patient’s family was informed about this limitation and offered referral, but they opted to continue treatment locally. The diagnosis was made based on classical clinical features: hemorrhagic crusting of lips, oral lesions, bilateral conjunctivitis, and the temporal association with amoxicillin exposure.

**Figure 2:**
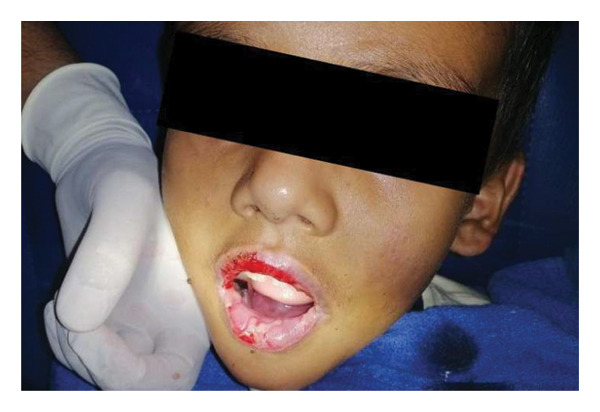
Operative debridement of devitalized oral epithelium revealing the underlying healthy oral mucosa.

The patient was admitted to the ward and started on intravenous (IV) fluids, IV ondansetron, IV pantoprazole, and IV paracetamol. The devitalized epithelium was removed via operative debridement revealing the underlying oral mucosa (Figure 2). A swab taken from the base of the crusted oral ulcers was sent for culture and sensitivity, which showed no growth of any organism. PCR for Mycoplasma pneumoniae was negative.

Dressing of the lip lesions was done every 4 h, and paraffin gauze was placed to prevent drying. With regular monitoring and dressing of the lesions, after 9 days, there was no evidence of lip ulcerations and bleeding.

**Figure 3:**
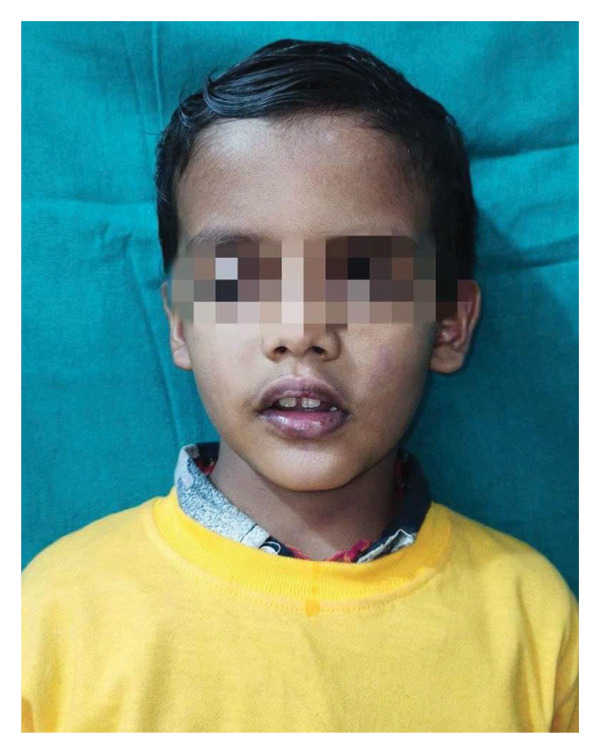
Follow‐up at 1 week.

Discharge medications included tablet zinc, tablet vitamin C, tablet paracetamol, and liquid paraffin ointment. Follow‐up after 1 week revealed complete resolution of symptoms with healed oral mucosa (Figure 3).

## 3. Discussion

SJS is an uncommon but severe mucocutaneous hypersensitivity reaction. Although its exact etiology remains unclear, it is most frequently associated with certain medications, infections, and, less commonly, malignancies [4]. SJS is a T‐cell‐mediated cytotoxic reaction that leads to keratinocyte apoptosis, causing extreme damage to the skin and mucous membranes. The immune response is initiated after a drug or its metabolite interacts with the immune system, resulting in the activation of CD8+ cytotoxic T‐cells. These cytotoxic T‐cells induce cell death of keratinocytes via pathways such as the perforin/granzyme or Fas/FasL systems [5]. The disease typically begins with erythematous macules on the skin, which may coalesce into blisters and eventually result in widespread epidermal separation. Mucosal involvement often affects the oral cavity and eyes [6]. The lesions may appear as purplish macules or atypical patches with indistinct margins, usually starting on the trunk before spreading to the extremities.

While oral involvement is frequently observed in SJS, isolated mucosal disease without cutaneous manifestations is a rare presentation. In the present case, a 6‐year‐old boy developed painful oral ulcerations and mucosal bleeding, without ocular, genital, or cutaneous lesions. These findings are consistent with mucosal‐predominant SJS, a subtype that occurs more often in pediatric populations. However, in the absence of skin lesions, the clinical picture may be confusing with other conditions such as herpes simplex infection, oral candidiasis, or pemphigus vulgaris [7].

This atypical form, also termed Fuchs syndrome, is most commonly associated with Mycoplasma pneumoniae infection, although drug‐induced cases have been reported as well [8]. Its immunopathogenesis is quite similar to classic SJS, involving infection‐ or drug‐triggered delayed hypersensitivity reactions, but without extensive epidermal separation [9]. Diagnosis is primarily clinical, based mainly on the presence of characteristic mucosal lesions and temporal association with drug exposure when relevant. Although histopathology can provide supportive evidence, it is not essential for diagnosis [10]. In our case, histological confirmation was not done; however, the typical mucosal findings, high index of suspicion, and rapid improvement after discontinuation of amoxicillin supported the diagnosis.

The absence of skin lesions often leads to diagnostic uncertainty emphasizing the need for careful evaluation. Management of SJS in Fuchs syndrome is mainly supportive: stopping the offending drug, fluid and nutritional support, and adequate pain control are important. In cases of suspected or confirmed infection by Mycoplasma pneumoniae, the administration of antibiotics effective against Mycoplasma is needed. Preferred antibiotics include clarithromycin and azithromycin, which are macrolides, followed by quinolones and tetracyclines. Tetracyclines, however, are best avoided in children below 8 years of age [11]. Systemic corticosteroids and IVIG are other possible adjunctive therapies to consider based on clinical severity [8]. Topical steroids also have shown efficacy in addition to systemic corticosteroids [12]. Beyond individual cases, public awareness of drug‐induced hypersensitivity reactions and routine screening strategies before initiating high‐risk medications can play an important role in minimizing complications and improving outcomes.

## 4. Conclusion

This report describes a rare case of mucosal‐predominant SJS in a child, presenting without the characteristic skin lesions. The absence of cutaneous involvement posed a diagnostic challenge. However, rapid clinical improvement following drug withdrawal and supportive management confirmed the diagnosis, even in a resource‐limited setting. This case highlights the importance of recognizing atypical presentations of SJS early, as timely diagnosis and intervention are critical in reducing complications and improving prognosis in this potentially life‐threatening condition.

## Ethics Statement

The report was written in accordance with the Declaration of Helsinki.

## Consent

Written informed consent was obtained from the patient for publication and any accompanying images. A copy of the written consent is available for review by the Editor‐in‐Chief of this journal on request.

The authors certify that they have obtained all appropriate patient consent form. The patient has given their consent for their images and other clinical information to be reported in journal. The patient understands that their names and initials will not be published, and due efforts will be made to conceal their identity.

## Conflicts of Interest

The authors declare no conflicts of interest.

## Author Contributions

Salina Paudel, Manoj Kumar Bhatt, and Shiv Kumar Chaudhary were involved in patient care. Sandhya Silwal, Abinash Parajuli, and Pramod Kumar Kafle were involved in investigations, resources, and writing–review and editing.

## Funding

This work did not receive any specific grant from funding agencies in the public, commercial, or not‐for‐profit sectors.

## Data Availability

The data that support the findings of this study are available on request from the corresponding author. The data are not publicly available due to privacy or ethical restrictions.
